# Development of self-efficacy skills of prospective music teachers in singing activity: insights of educators

**DOI:** 10.3389/fpsyg.2025.1685205

**Published:** 2025-10-27

**Authors:** Guanhua Bi, Asta Rauduvaitė

**Affiliations:** Academy of Education, Vytautas Magnus University, Vilnius, Lithuania

**Keywords:** self-efficacy, prospective music teachers, singing activities, emotional regulation, teacher education

## Abstract

This study explores how self-efficacy is developed and expressed in prospective music teachers through singing-related coursework in Chinese higher education. Using a qualitative research design, data were collected through semi-structured interviews with eight university music educators who had experience teaching solfeggio, vocal music, choir, and Chinese opera. Qualitative content analysis revealed that self-efficacy in prospective music teachers is closely related to confidence, emotional regulation, collaboration, adaptability, and other essential skills for professional growth. Educators highlighted the importance of designing manageable singing tasks, encouraging peer observation, providing targeted feedback, and fostering a supportive and emotionally safe learning environment. The findings suggest that by systematically integrating self-efficacy enhancement strategies into music education curricula, educators can promote the holistic development of prospective music teachers. These insights contribute to practical implications for curriculum design, teacher preparation, and the ongoing advancement of music education in the context of contemporary challenges.

## Introduction

Over the past decade, music and arts education has undergone notable shifts in pedagogy and course design, including wider integration of digital tools and reconfiguration of ensemble and studio practices, with implications for motivation and self-efficacy (Schiavio et al., 2020; Lee et al., 2021; Gill et al., 2022; Zhao and Cao, 2023). Therefore, since ‘teaching’ is framed not merely as conveying ideas but as preparing culturally responsive, inclusive practitioners through authentic immersive experiences (Achieng, 2023; Fuelberth and Woody, 2023). Rather than being framed as mere transmitters of knowledge, contemporary teachers are expected to facilitate learning and foster students’ self-regulated learning and emotion management, with pedagogical choices informed by self-efficacy considerations (Zhu, 2020; Regier, 2022; Muñoz, 2021). In such a context, teachers are challenged with requirements of a more advanced level of professional confidence, flexibility in pedagogy, self-regulation, and reflective practice needs, which, in turn, justifies the necessity of modern teacher education programmes to make their priorities of developing these capacities explicit (Tschannen-Moran and Hoy, 2001; Āboltiņa et al., 2024).

In this changing context of education, the concept of self-efficacy that was initially propounded by Bandura (1977, 1986) has taken a central position in teacher development studies. Self-efficacy is embedded in the social cognitive theory of Bandura (1977), which refers to the confidence of an individual in terms of performing a set of activities that can deliver desired outcomes (Zimmerman, 2000; Farmer et al., 2021). Similarly, the empirical evidence in teacher education demonstrates that strong self-efficacy leads to higher levels of motivation, more positive emotionality, greater commitment in terms of cognitions, and behavioural perseverance (Tschannen-Moran and Hoy, 2001; Sun, 2022). Teachers with higher self-efficacy tend to use unconventional teaching approaches, encourage classroom dynamics, embrace student initiative, and make them resilient to failure-related setbacks (Seçkin and Başbay, 2013; Shah, 2023). Conversely, lower self-efficacy is associated with higher anxiety, avoidance of challenging tasks, and increased risk of burnout (Ma et al., 2021).

These performance-based platforms provide the nurturing environment in which Bandura’s four sources of self-efficacy are activated. Self-efficacy is shaped by four interconnected sources: mastery experiences; vicarious experiences; verbal persuasion, and physiological/emotional states (Bandura, 1977; Usher and Pajares, 2008; Scoloveno, 2018). Mastery experience is often considered the primary source of self-efficacy (Bandura, 1977; Royo, 2014; Warner and Schwarzer, 2017; Hier and Mahony, 2018). That is, successfully overcoming challenges and repeatedly building confidence, which supports further action (Clark, 2019; Bhati and Sethy, 2022). In turn, vicarious experiences concern learning based on the observation of successes and failures of others; they are particularly effective among novice teachers or students who lack their personal records of previous success (Warner and Schwarzer, 2017; MacAfee and Comeau, 2020). Self-efficacy increases when trusted individuals provide specific, positive feedback. Conversely, it may be diminished (Clark et al., 2014; Waters, 2020; Legette and Royo, 2021). Finally, subjective assessment of physiological and emotional conditions of people (such as anxiety, excitement, or tiredness) also influences efficacy beliefs and directs them to respond to the teaching or learning problem (Brooks, 2014; Sander, 2020; Han et al., 2022).

Rather than acting independently, the four sources operate as interacting processes. Vicarious experience and verbal persuasion frame how students interpret their mastery attempts (perceived attainability, standards), physiological/emotional states moderate the impact of modelling and feedback, and mastery outcomes then feedback to recalibrate the credibility of models and feedback, forming a ‘forethought-performancereflection’ cycle through singing-related coursework (Bandura, 1977; Usher and Pajares, 2008; Lu and Suo, 2020; Liu, 2021).

The importance of self-efficacy in teacher education has been widely documented. Teachers with strong self-efficacy become highly involved in their professional growth. They are more open to the changes in education applied, and foster positive student outcomes (Klassen and Chiu, 2010; Biasutti et al., 2020; Doménech et al., 2024). This relationship is reciprocal: a teacher with the feeling of self-efficacy develops student motivation and resilience, and vice versa, the positive engagement of students makes a teacher more confident in his or her skills (Björklund et al., 2020; Bergee and Grashel, 2002; Cohen and Panebianco, 2020). In this article, “educators” refers to university music teachers (our interview informants), whereas “prospective music teachers” refers to the target group. We analyse educators’ narratives to understand how these students’ self-efficacy is expressed and developed in singing-related coursework; we do not directly measure students’ self-efficacy. Teachers’ self-efficacy is discussed only as an antecedent shaping feedback, demonstration, and task sequencing. Building on this reciprocal pattern, research shows that teachers’ self-efficacy is positively correlated with their emotional well-being, job satisfaction, and professional identity (Xu, 2022; Zelenak, 2020; Concina, 2023; Ma et al., 2021).

Within music education, self-efficacy is of distinctive importance. As opposed to most fields of studies, music education cannot exist without public performance, emotional expression, and cooperative artistic creation (Hallam, 2010; Burnard et al., 2018; Paolantonio et al., 2023). Since the process of music education occurs in a naturally performative, affective, and social environment, students (prospective teachers) are frequently exposed to performance anxieties, recover from setbacks, and seek to find confidence in their musical voices (Fancourt and Finn, 2019; Wang, 2022; Morgan, 2019; Vaizman and Harpaz, 2022). Therefore, it is up to music teachers to develop not only technical skills and mastery of concepts but also creativity, emotional resilience, and cooperation skills needed in an ensemble (Osborne and McPherson, 2018; Sander, 2020; Gill et al., 2022). These skills both depend on and reinforce self-efficacy, operating through mastery experiences, vicarious experiences, verbal persuasion, and physiological/emotional states (Bandura, 1977; Schunk and DiBenedetto, 2021).

In Chinese higher music education, singing-related coursework (Solfeggio, Vocal Music, Chinese Opera and Choir) places prospective teachers in performative and affect-laden settings (Guan, 2023; Wang, 2022). Typical challenges include breath support at higher phrases, smooth register transitions, intonation/timing/blend in choir, language-specific diction, and juried-exam/recital anxiety (Liu, 2018; Yang and Welch, 2023).

This study has its theoretical background in the social cognitive theory developed by Bandura, which defines self-efficacy as one of the key constructs that determine motivation, learning strategies, and persistence in the profession (Bandura, 1977; Schunk and Pajares, 2009; Michaud, 2023). Special attention was paid to Bandura’s four sources of self-efficacy (mastery experiences, vicarious experiences, verbal persuasion, and physiological/emotional states), as the canonical lens in music teacher education (Tschannen-Moran and Hoy, 2001; Pan, 2020). Guided by this framework, we used a theory-informed interview guide to ensure coverage in questioning, while coding and categorization were conducted inductively; the theoretical lens is used for interpretation rather than as a coding frame.

According to the Ministry of Education of the People's Republic of China (2006) curriculum scheme for the undergraduate Music Education (teacher-training) major, singing-related courses (Solfeggio, Vocal Music, Chinese Oprea, and Choir) account for approximately 36% of required courses (4/11), 40% of total contact hours (414/1,170), and 38% of total credits (23/60), which underscores their curricular centrality. These courses also provide frequent public-performance opportunities (e.g., recitals, juried exams, outreach concerts), increasing students’ exposure to evaluative feedback, peer collaboration, and emotional self-regulation. As a result, singing coursework concentrates Bandura’s four sources of self-efficacy: sustained practice and stage performance (mastery), observing lecturers and peers (vicarious experience), targeted feedback (verbal persuasion), and managing arousal on stage (physiological/emotional states; Tian, 2019; Wang, 2020; Wei, 2023; Xu, 2025). These are precisely the conditions in which self-efficacy is expressed and developed; hence singing is an apt domain for the present inquiry.

**Research question**: How the expression and development process of prospective music teachers’ self-efficacy skills occurs in university studies that facilitates their engagement in the singing practice.

**Aim of the research**: To theoretically and empirically reveal the possibilities for developing prospective music teachers’ self-efficacy skills in singing activity.

**Objective of the research**: To reveal and systematise the methods for developing self-efficacy skills of prospective music teachers in singing activity during their university studies.

Although the significance of self-efficacy as a central factor in the formation of music teachers is fairly well known, the processes in which self-efficacy is cultivated in a singing-related lecture setting remain insufficiently explored. Over the past several years, the literature has focused on limited post-hoc quantitative surveys or self-report instruments, neither of which is effective enough to capture the dynamic, context-specific, and interpersonal factors at play in actual classroom and rehearsal settings (Legette and Royo, 2021; Paolantonio et al., 2023; Wang, 2022). Recent scholarship has been committed to redirecting empirical investigation towards qualitative contextually sensitive forms of investigation that explicate the process by which self-efficacy occurs, manifests and persists through the lived experiences of students, teachers and peers (Pan, 2020; Ye, 2023; Sander, 2020).

To address these gaps, the present study uses semi-structured interviews with eight educators from normal universities in China, each possessing extensive experience in teaching core singing-related courses. This study explores how educators perceive and nurture self-efficacy in prospective music teachers during singing-related classes. It examines the behavioural and emotional signs of self-efficacy, the key factors that influence it, and the effective teaching strategies educators use. Through qualitative analysis, the research aims to deepen theoretical understanding and offer practical insights for improving curriculum design and teacher education.

## Methodology

### Study design

This study was carried out using a qualitative research design in order to understand the process of developing and expressing self-efficacy among prospective music teachers in Chinese higher education with respect to singing-related lectures. Participants were eight university music educators (key informants) who teach core singing-related lectures (Solfeggio, Vocal Music, Chinese Opera, Choir) with the eight. The empirical data were collected via semi-structured interviews with eight university music educators. The semi-structured guide covered typical domains in singing coursework: (i) opportunities for progress and public performance, (ii) teacher/peer demonstration and modelling, (iii) feedback and affirmation practices, and (iv) strategies for managing performance arousal. These domains ensured coverage in questioning; they did not constrain the inductive coding.

This approach was chosen because educators, as instructors of core singing-related lectures, possess unique insights into the pedagogical processes, challenges, and opportunities associated with fostering self-efficacy in prospective music teachers (Bresler, 2021; Marques and Mateiro, 2024). We analysed educators’ accounts and limited our claims to educators’ perspectives; we did not directly measure students’ self-efficacy. Bandura’s sources guided the interview protocol only; no *a priori* theory-driven categories were imposed during coding.

Qualitative inquiry was chosen on the basis of its capacity to reflect the multifactorial and subject-situational character of self-efficacy formation, the applicability of which to artistic, performative, and emotionally loaded spheres like music education is evident (Tracy, 2020; Goodman-Scott et al., 2024; Makateng and Mokala, 2025). By collecting and analysing reflective narratives of educators, the present study explores the behavioural and emotional aspects of self-efficacy development in prospective music teachers, as understood from the perspective of teacher interviewees in the context of singing-related lectures.

### Participants

Eight educators from eight normal universities in China were interviewed using the purposive sampling. All participants actively taught fundamental singing-related lectures (Solfeggio, Vocal Music, Chinese Opera, and Choir) and had significant experience in dealing with and training prospective music teachers, ensuring both depth of pedagogical knowledge and contextual relevance. The gender distribution included both male and female teachers. All interviewees were assigned codes (E1-E8), which are used to present direct quotations and findings. Inclusion criteria were: (i) teaching at least one core singing-related lecture; (ii) ≥ 10 years of university-level teaching; and (iii) responsibility for lecture design and assessment (e.g., juries/recitals) across multiple cohorts. We interviewed educators as key informants because the study aimed to identify how prospective music teachers’ self-efficacy is developed in singing-related coursework; these instructors design and enact the relevant practices across multiple cohorts, providing a broader comparative view than single-student self-reports. This type of selection strategy guaranteed that the data could be pedagogically important and based on actual educational practice (Etikan, 2016; Bouncken et al., 2025).

The present study focused on third- and fourth-year undergraduate students (prospective music teachers) enrolled in music teacher education programmes. This period is a critical transition in which students move from learners to novice educators, integrating advanced musical expertise with pedagogical training and school practicum (Fisher et al., 2021; Wang et al., 2022). By Years 3–4, prospective music teachers typically undertake capstone juries/recitals, ensemble leadership, and practice-teaching (students’ own micro-teaching and school practicum or internship), and are expected not only to refine artistic skills but also to consolidate a professional identity as music educators. Consequently, the expression and development of self-efficacy are more observable at this stage: juries/recitals and successful run-throughs provide mastery experiences; studio and ensemble work afford vicarious modelling; mentor/peer calibrated feedback supplies verbal persuasion; and public performance requires arousal regulation (physiological/emotional states). Early-year cohorts have more limited exposure to these performance- and practicum-based contexts and were therefore not the analytic focus of this study.

Sample size followed information power and saturation principles: recruitment ceased at eight when iterative analysis indicated no new codes or subthemes (thematic redundancy).

### Data collection

Semi-structured interviews with eight educators from eight universities were used in the research to explore how prospective music teachers develop self-efficacy in singing-related lectures. A semi-structured interview is a well-established qualitative method that is based on open-ended questions and a flexible framework, which allows for an in-depth exploration of research participants’ thoughts, feelings, and experiences (Adeoye-Olatunde and Olenik, 2021; Buys et al., 2022; Ruslin et al., 2022).

The interview guide was constructed using four sources of self-efficacy by Bandura, and previous research findings in music teacher education (Bandura, 1977; Butler, 2024; Ge, 2024). Semi-structured interviews followed six core prompts: 1. How educators define and understand the concept of self-efficacy in prospective music teachers; 2. Based on the educators’ experiences, the factors that most influence the self-efficacy of prospective music teachers; 3. How they design tasks to help prospective music teachers build self-efficacy during their lectures (Solfeggio, Vocal, Chinese Opera, Choir); 4. How they promote prospective music teachers’ self-efficacy by allowing them to observe the educators’ modelling in their courses; 5. How verbal encouragement can be used to increase prospective music teachers’ self-efficacy; 6. How they help prospective music teachers maintain positive emotional and physiological states. Questions were flexibly sequenced with probes as needed. The full interview guide is provided in Appendix S1.

Before data collection, all participants were informed about the purpose of the research, assured of confidentiality, and provided written consent. The interviews were conducted in China. Interviews were in Mandarin and verbatim-transcribed by the first author; a bilingual assistant audited a subset and English excerpts. We used a bilingual codebook (Mandarin→English) for team analysis; the second author reviewed the English materials, and differences were resolved by discussion. Face-to-face or secure online channels were selected based on the participants’ availability. Each session lasted between 35 and 55 min. All interviews were conducted with the consent of the participants and recorded and transcribed for analysis.

To ensure depth and authenticity, the interviewer participants reflected on specific classroom practices, provided descriptive examples, and explained their pedagogical rationale and emotional connection to their students. Questions were flexibly sequenced, and then probe questions were asked depending on the response of the participants. The non-rigid structure permitted probing and flexibility to the environment and teaching philosophies of each interviewee which contributed to supplementing the data with a realistic educational knowledge base and efficacy experiences.

This statement clarifies the researchers’ neutral relationship with participants and the use of ethical safeguards. It was added to ensure transparency, reflexivity, and trustworthiness, addressing reviewer concerns about possible conflicts of interest or undue influence.

This clarification specifies that, while no institutional ethics committee approval was obtained, the study complied with ethical standards in qualitative research by securing voluntary informed consent, ensuring confidentiality, and using pseudonyms. It was added to enhance ethical transparency in response to reviewer concerns.

Participants were invited through departmental contacts and professional networks. The first author personally identified and selected experienced educators to ensure relevance. Participation was voluntary and uncompensated.

### Data analysis

In this study a qualitative content analysis was applied to analyse and organise the data drawn after semi-structured interviews. Patterns, categories, and meanings in textual data were identified through a systematic method called qualitative content analysis. It can help researchers understand manifest and latent matter in communication processes (Strecher et al., 1986; Rampin and Rampin, 2021). Such an approach is particularly well-suited for educational and psychological studies as it accommodates rich, complex data without undermining the contextual meaning of the participants’ narratives (Bingham, 2023; Kuckartz and Rädiker, 2023; Nicmanis, 2024).

The first author conducted inductive qualitative content analysis following Mayring’s (2021) rule-guided procedures. The author read the transcripts repeatedly, assigned open codes to meaningful segments, compared and merged similar codes, and grouped them into subthemes and broader themes. No *a priori* theory-driven categories were imposed; all categories were data-driven.

All interviews were audio-recorded and transcribed verbatim. Transcripts were then segmented into meaning units (sentences/clauses expressing a single idea). The first author retained segments that spoke directly to the research question, such as: (a) educators’ understanding of self-efficacy; (b) factors influencing prospective music teachers’ self-efficacy; (c) educators’ practices (modelling, feedback/affirmation, task design); and (d) singing-lecture contexts (rehearsals, run-throughs, juries/recitals). Small talk and logistical remarks were excluded. No a priori theory-based filters were applied; disconfirming/negative cases were retained and coded. This procedure focuses the analysis on relevant themes while minimising selection bias (Song et al., 2021; Ackermann and Merrill, 2022; Ningi, 2022).

Codes were compared within and across interviews; near-duplicates were merged, overly broad codes split, and boundaries refined. Conceptually related codes were grouped into subthemes and then elevated to a small set of overarching themes with inclusion/exclusion rules. Coding was conducted by the first author; given the inductive, reflexive design, no inter-coder agreement statistics were computed. Dependability was enhanced via a code-recode check on two interviews (with refinements to code definitions as needed) and co-author peer-debriefing at two checkpoints (after interviews 3 and 6), reviewing the codebook and theme map.

The first author maintained (i) a dated code log (code name, definition, exemplar quotation, interview ID), (ii) decision memos documenting merges/splits/renaming and inclusion rules, and (iii) a versioned theme map with timestamps. The first author ran internal checks after each transcript, at two checkpoints (after interviews 3 and 6), and a final pass before write-up; no external auditor was engaged. Typical boundary issues (e.g., targeted feedback vs. affirmation; teacher vs. peer modelling) were resolved by refining definitions and, where appropriate, dual-coding overlapping excerpts (Nowell et al., 2017; O’Kane et al., 2021).

## Results

This study collected the experiences of 8 educators from 8 normal universities. These experiences covered the teaching process from vocal teaching, choir, solfeggio and Chinese opera. The primary intent of this study was how to promote the self-efficacy of prospective music teachers with a focus on the strategies educators use to strengthen their students’ self-efficacy during their various teaching activities and practices.

### Definitions and understandings of self-efficacy

At the beginning of the interview, it was important to identify *how educators define and understand the concept of self-efficacy in prospective music teachers.* Respondents noted that self-efficacy plays a central role in helping prospective music teachers overcome challenges and build confidence. Educators noted that a high level of self-efficacy influences emotional well-being and expressiveness, helping to develop the adaptability and collaboration necessary for prospective music teachers to succeed in learning and performing.

Table 1 shows that the self-efficacy of prospective music teachers refers to the students’ confidence in their skills to face challenges, adapt to different types of music, and take on various roles in performances. It also emphasises their collaboration skills with others while more effectively managing and expressing their emotions and musical skills. As Educator E5 mentioned, students in higher education already have a basic foundation in music, and self-efficacy is a key factor in helping them get to the next level. It is common for students to feel pressure when they encounter difficulties in learning and performance, such as ‘complex rhythms’ in solfeggio or ‘high notes’ in vocal practice. Self-efficacy helps students build confidence in their skills to face these challenges and encourages them to say to themselves ‘I can do it.’ Such an attitude motivates a student to seek alternative options to find a solution to problems and move at a more increased pace achieved by relentlessly engaging in practice. Educators also emphasise that their task is to make students gain this confidence through practice so that they become successful in their future musical careers.

**Table 1:** Definitions and understandings of self-efficacy in prospective music teachers.

Themes	Statements of respondents
Confidence and overcoming challenges	*Self-efficacy in higher education refers to a student’s trust in own competences and motivation not to give up. When students are faced with a technical challenge, such as ‘how to sing high notes,’ they must first have to believe that they can ‘sing.’ It’s the ones who have confidence in themselves that are more willing to put in the time and effort to practice and find different ways of doing things. My job is to help students build that confidence in singing so that they believe they can do it. (E3); It’s like a fire in the hearts of the students; it burns their confidence in their skills. It also serves as an internal motivation that motivates students to improve their musical learning and practice. For example, in solfeggio lessons, it is quite common for students to feel stressed because of tempo or pitch problems. However, if they believe they can solve these problems through practice, their progress will be faster. (E1)*
Emotional regulation and expressiveness	*Students must regulate and express their emotions according to different role needs in Peking Opera, students with high self-efficacy are more likely to do this, it’s not something you can touch or see, but it makes you believe that you are the character and can move the audience. It allows you to communicate the emotions of the character on stage confidently. (E6); Self-efficacy is like an internal metronome for music learners; it drives their ‘emotional tempo’, influences their emotional expression. It allows students to believe that they can express every symbol on the sheet music well and sing every note accurately. (E2)*
Collaboration and teamwork	*Students with high self-efficacy in chorus classes show a greater willingness to cooperate with others to ensure the overall harmony and performance of the team. When they encounter difficulties in rehearsal, they usually first reflect on where they need to improve and find ways to communicate effectively with their peers to solve the problem. (E7); My students often perform duets or small ensembles. When students believe in their skills. They are more willing to communicate with their peers, more eager to help others, show empathy, and a strong sense of teamwork. (E5); Some students may not have the best voice, but I can sense their confidence and motivation. This positive energy influences their peers both in solo performances and in choir settings. They are good at motivating themselves and also cheering on their peers, encouraging them to perform better. (E8)*
Learning agility and adaptability	*As a solfeggio lecturer, I understand self-efficacy as the students’ confidence in their skills to trust and adapt to their understanding of complex pitch, rhythm, and music theory. (E1); Students with high self-efficacy always show great adaptability and learning agility, and are able to quickly understand the character of a new role and adjust their singing style to be flexible in rehearsals and performances. (E4)*

Self-efficacy is also extremely significant where emotion regulation and expression are concerned. Educator E2 likened it to an ‘internal metronome’ that controls one’s ‘emotional rhythm.’ Music students face various pressures during the performance, such as different performance environments, different audiences, personal status on the day of the performance, or unexpected during the performance. They need to believe in their skills to control and regulate their emotions to better and more freely express the feelings the composer wants to convey.

In terms of teamwork, prospective music teachers’ self-efficacy is understood by Educator E8 as a positive energy and motivation. These students may not have the best voice or the best musical skills, but their self-confidence and positive attitude affect others in the team. The interviewees noted that students with high self-efficacy are more willing to collaborate with others, including showing empathy and teamwork, self-reflection, and encouraging their peers. They believed in their skills to help themselves and their peers improve their performance.

Last the educators highlighted the fact that self-efficacy plays an important role in developing learning agility and adaptability. As prospective music teachers, students should be flexible in terms of new music and styles, complex rhythms, and varying performance demands. As Educator E4 mentioned, students with high self-efficacy show more adaptability and learning agility during practices and performances. The concept of self-efficacy is known to teachers as an important consideration for prospective music teachers, and their goal is to help students improve their self-efficacy to prepare them for their future careers as music educators.

### Factors influencing self-efficacy

After understanding how music professors define the self-efficacy of prospective teachers, the second interview question was to explore, *based on the educators’ experiences, the factors that most influence the self-efficacy of prospective music teachers.* Respondents state that practice-teaching experience, peer collaboration, constructive feedback, and the mastery of musical skills are key contributors to building self-efficacy.

Table 2 shows that the interviewed educators suggested important factors affecting prospective music teachers’ self-efficacy based on their own experiences. These are practice-teaching experience, constructive feedback, peer collaboration and observation, mastery of musical skills, and emotional regulation and adaptability.

**Table 2:** Factors influencing self-efficacy in prospective music teachers.

Themes	Statements of respondents
Practice-teaching experience	*It’s challenging for students when they step up to the podium on their own to teach others. But in my opinion, practice is the best way to increase their self-efficacy. In practice they discover their strengths and weaknesses, meet a wide* var*iety of students, and ask a wide variety of questions. But I believe they will grow with each solution. (E1); The more success you have, the stronger your self-confidence will be. This is what I have always believed. So in my teaching, I will lead students to experience more successful experiences to help them accumulate self-efficacy. (E6); The actual classroom and teaching experience, especially if you gain a sense of control in the classroom, is a huge boost to your self-efficacy. (E7); Once I let a student conduct a short choral exercise. At first he was very nervous about making mistakes, but when he gradually guided the other students to complete the exercise, his whole state became more relaxed and his conducting gestures became more precise and natural. Afterward, he said to me with great confidence, ‘I can actually teach this part well, teaching is not as hard as I thought.’ This kind of successful experience is very important for their self-efficacy. (E8)*
Constructive feedback	*My teachers had the greatest influence on me and recognising me would have been a great boost to my beliefs about teaching! So I think it’s important to give prospective music teachers positive, encouraging comments in order for them to grow in self-efficacy. (E2); When starting a new job, the guidance of experienced colleagues and the process of getting to know the students is very important. Feedback from your supervisors and students is what most helps boost your self-efficacy. (E7) When I first started conducting choir rehearsals, one section kept struggling with rhythm, even after I had guided them several times. I felt a sense of helplessness and even started to doubt myself. But that section of students started reassuring me, saying, ‘It’s okay, teacher. Let us try again, we can definitely do better.’ Their comforting words warmed my heart. This kind of support is essential for the self-efficacy of future music teachers. (E8)*
Peer collaboration and observation	*When one of the students in the group exercise saw his peers successfully lead the other students through a complex musical segment, he immediately tried the same approach to lead the students through the exercise and it went very well. This confidence gained by observing others often helped them progress more quickly. (E2); Vocal music is relatively abstract to teach. Students must learn to appreciate the various resonant positions of the body, not just by being told by the teacher, but also by listening to other people singing, observing their methods and techniques, and learning from their strengths to make up for their weaknesses. (E3); Collaborate with experienced colleagues, be humble in your collaboration, and observe and reflect on the strengths of others’ teaching that you can learn from. Listening to other music professors’ classes will give you a different perspective on teaching, which will help you grow in your own confidence in teaching. (E6)*
Mastery of musical skills	*I think as a teacher you need to keep improving whether you recognize you have the necessary skills or not. Constantly absorbing new knowledge to pass on to your students. (E1); Professional knowledge base is critical to prospective music teacher self-efficacy. Without an overwhelming amount of knowledge reserve, one’s confidence will definitely be affected. Solid music expertise and pre-lesson preparation will enable me to know how each lesson should be conducted. (E2); The biggest influence on confidence is the feedback you get from your students in class, which depends on how well you have mastered the musical skills you are trying to teach and how well you have prepared in advance of the lesson. (E4)*
Emotional regulation and adaptability	*I have seen many newly employed teachers get emotional when something unexpected happens in class, such as when students do not get what they want to say or disagree with their teaching methods, which affects the whole teaching process. I would like to tell them that mistakes in teaching are not scary; what matters is how you face them and adjust them in time. (E3); Prospective music teachers should learn to control and regulate their emotions while they are still students. For example, public performances in the classroom or on stage are good opportunities for them to practice regulating their emotions. As a result, they will be able to remain calm in future classes, stop letting small mistakes affect overall teaching, and gradually show more control. (E6)*

Almost all educators mentioned the first factor of practice-teaching experience in their responses, that is teaching experience, especially successful teaching experience and noted that this was critical to their self-efficacy. They gained one of the most direct experiences in successful teaching, which allowed them to experience the various situations in the teaching process. As Educator E1 pointed out, there are various problems, various types of students that the teacher has to face in the teaching process. Then the problem-solving process to find their own strengths and weaknesses, so as to improve to grow. Practice is the best way to test what you have learnt, and the teachers mentioned that their self-efficacy increases every time a problem is solved.

The role of mentor feedback, colleagues, family and students feedback is critical in enhancing self-efficacy. Encouraging and positive responses help warm up teachers and feel supported, making them and more confident as far as their own teaching skills are concerned. Educator E2 emphasised the importance of encouragement from the prospective teachers’ own instructors, suggesting that positive and supportive comments help develop their self-efficacy. Similarly, Educator E8 recalled an anecdote of a student giving him a soothing word that allowed him to overcome the feelings of frustration after a challenging choir rehearsal. Such experience implies a positive impact of positive feedback related to music teacher’s self-efficacy.

Another factor that educators referred to frequently was peer collaboration and observation. A significant number of the interviewees explained how much they have learnt by observing their means of teaching through their colleagues, and this has helped them in their practice. Educator E2 remembered that the confidence they received by observing others usually allowed them to advance. Educator E6 pointed out the need to collaborate with more experienced peers and consider their pedagogical assets and to be humble, which will eventually help to induce growth in the sense of self-efficacy building. Being surrounded by others and learning through observation not only grant practical ways of doing things but also reinforce the belief that people can achieve similar successes.

The fourth factor is the mastery of musical skills, which is the basis for developing confidence in teaching. Being a teacher, a solid foundation is needed to constantly improve oneself by absorbing new knowledge and setting an example for students. The educators agreed that a solid foundation of musical knowledge and technical skills was vital for prospective music teachers. Educator E4 emphasised that instructors’ strong musical command and thorough preparation enable more specific, timely, and encouraging feedback, thereby more effectively enhancing students’ self-efficacy.

The educators also pointed at emotional control and flexibility as the important ones in retaining self-efficacy. They discussed their life experiences in coping with unpredictable circumstances, controlling their emotions during stress, and how to adjust to new surroundings. Educator E3 stated that prospective music teachers should learn to face mistakes with a positive mindset and make timely adjustments. Educator E6 added that it is important to seize the opportunity of public performances to exercise to learn to regulate emotions when they are students.

### Task-design strategies to build self-efficacy

Having explored the factors influencing prospective music teachers’ self-efficacy, educators were asked *how they design tasks to help prospective music teachers build self-efficacy during their lectures (Solfeggio, Vocal, Chinese Opera, Choir).* Their responses highlighted the importance of designing tasks that start with minimal demands and gradually increase, allowing students to build on small successes. They also emphasised the value of group learning and maintaining task variety to boost student engagement.

Table 3 shows how music teachers design tasks in their courses to build prospective music teachers’ self-efficacy. Most educators mentioned that ‘gradual progress’ is the core idea of task design. Educators break down difficult tasks such as complex rhythmic, big melody jumps, or singing ‘high notes’ into small, more manageable parts, and gradually increase their complexity as students gain more mastery and understanding. This gradually increases the student’s confidence in their skills. Just like what Educator E2 did in the solfeggio class, he led students to start with simple scales and sang with them to support them, then gradually reduced his participation and let students sing on their own. The accumulation of these ‘small successes’ made students realise that they are capable of handling complex music tasks on their own, increasing their self-efficacy. Educator E3 shared his experience as a vocal teacher, where many of his students had a fear of ‘singing high notes’. Faced with this situation, E3 would start practicing with the lower and middle vocal registers, which not only cultivated students’ stability in the middle range but also did not make them feel frustrated because it was too difficult. Sometimes, students unknowingly reach high notes naturally. These methods allow students to feel their progress after completing each small task and gradually improve their self-efficacy.

**Table 3:** Task design strategies to build self-efficacy.

Themes	Statements of respondents
Gradual progression	*I would have them start by singing a simple C major scale, and at first I would sing it with them. Then, slowly reduce my involvement and let them do it on their own. When they realise that they can do it on their own, they get that look on their face that says, ‘Oh, I’ve done it.’ It’s those little moments of success that make them feel like they are up to the challenge. (E2); Most students feel ‘afraid’ when approaching high notes. This is a common fear - they worry that they will not be able to sing the high notes, that they will be hoarse, or that they will make a fool of themselves in front of their classmates. I know exactly where they are coming from, so vocal warm-up exercises tend to start in the relatively easy bass register and work towards the middle register in to the relatively difficult high register, reminding them in the process to focus on keeping their breath supported and keeping their vocal placement rather than telling them which note they have already sung, to the extent that they do not realise they have already sung it high enough. (E3)*
Collaborative learning	*I also arrange some small solfeggio competitions in class, such as dividing them into two groups and asking them to take turns singing some short piece. Or let each student sing a line of the melody, and then the next student should sing the next line. The competition does not have to be complicated but will make them more motivated to participate. Everyone will cheer each other on and this atmosphere can especially help them build self-efficacy. (E2); ‘sectional rehearsals’ are quite common in choirs. This is especially suitable for choirs who are just starting to learn new works. During rehearsal, I will let each part rehearse separately for a period of time, so that they can fully master their own melody and rhythm. When they find confidence in their own voice, I will gradually blend the parts together. This practice reduces the anxiety of students who face the pressure of multiple voices at first. When they find that the melody of their part is already well sung, a look of ‘I can do it’ will appear on their faces. (E7); The essence of choir is teamwork. I often use the method of “peer teaching.” For example, the soprano will lead the singing and the other parts will be responsible for imitating their tone and rhythm. This method not only makes leading students feel their importance in the choir but also allows imitating students to learn to focus more on the unity of timbre. (E8)*
Task engagement and variety	*Make them interested first so they will be willing to learn. (E1); Vocal music teaching is very personalised and we need to choose the right practice method for each student. We should try to use as many different methods as possible so that they do not feel bored. (E4); Sometimes, I would ask male and female students to swap roles, with male students playing the role of women and female students playing the role of men. This not only adds to the fun but also allows them to have a deeper understanding of each other’s roles, which is helpful for their future cooperation. (E5); When singing in chorus, it is easy to be disturbed by other parts in pitch, especially students singing the low part, because they usually do not sing the main melody. I will design some “competitive exercises.” For example, let one part keep the sustained sound and the other part practice singing the melody based on this. (E7)*

The educators highlighted the advantages of group learning. Educator E2 described the joyful and supportive learning atmosphere created by students from different groups during group competitions. Such group exercises are most common in choral rehearsals. Educator E8 stated that the essence of choral singing is teamwork. Therefore, more collective factors should be considered when designing tasks. For example, ‘sectional rehearsals’ and ‘peer teaching’ allow students to better master the melody and rhythm of their own parts, so that they believe that their voices are in the team, thereby strengthening their self-efficacy.

The participants also pointed out that increasing task variety helps keep students interested in learning activities, which improves their engagement and contributes to the development of self-efficacy. E1 and E5 shared examples from their solfeggio and Chinese opera classes, such as rhythmic clapping exercises and role-swapping activities. Educator E7 stated that he designed a ‘competition exercise’ based on the situation that low-voice students tend to be out of tune and frustrated in choir rehearsal. These diverse and personalised exercises designed by educators for different students increase students’ motivation to participate in learning tasks, which is crucial to maintaining students’ enthusiasm for learning.

### Modelling and vicarious learning

An important source of self-efficacy is vicarious experiences that involve observational learning. Educators were asked *how they promote prospective music teachers’ self-efficacy by allowing them to observe the educators’ modelling in their courses*. Respondents indicated that creating a supportive environment that combines multiple forms of modelling through dynamic demonstrations from easy to difficult, realistic failure demonstrations, and various forms of teacher-student interaction. Successful demonstration of a teacher provides students with a clear and specific role model to follow which is very motivating, encourages students to be better confident of their strengths and offers vicarious experiences to increase their self-efficacy.

Table 4 presents the strategies educators use to improve the self-efficacy of prospective music teachers by allowing them to observe and engage in their modelling. Responses were classified into four subcategories: gradual progression, active engagement, focus on the process, and error-based learning. Educators usually demonstrate the reduction of difficulty in steps so that students understand that even difficult techniques can be broken down and achieved step by step. As Educator E4 explained, students gradually overcome their fear of ‘high notes’ and build up their confidence in completing the task by observing teachers breaking down a complex task into manageable steps. Similarly, Educator E7 gave an example of how a simple voice of a melody should be learnt at first and then only the complete harmony is introduced starting with one voice. All these structured ways help to make things easier and to gain confidence in skills of students.

**Table 4:** Strategies for promoting self-efficacy through observing educators’ modelling.

Themes	Statements of respondents
Gradual progression	*Many students are afraid to sing ‘high notes,’ which leads to a decrease in their self-efficacy. My demonstration usually starts with a simple breathing exercise to help them breathe correctly, and then I will explain to the students as I sing the high notes, ‘See, my high notes are not forced out, but are achieved naturally through breathing support and resonance adjustment.’ (E4); I visualise and make understandable complex elements of performance through demonstration and help students increase their self-efficacy through imitation and practice through step-by-step guidance. (E5); I start by demonstrating a single voice part of a melody, breaking down the rhythm and emphasis of each phrase. Then, I gradually add the other parts, building up to the complete harmony. Through observing and mimicking my step-by-step demonstration, students begin to understand the logic of choir singing and gain greater confidence in their own skills. (E7)*
Active engagement	*After completing a demonstration, I often invite students to be the “second demonstrator” to show my method to other students. For example, I will choose a student with good expressiveness and ask him to imitate my melody and lead others to practice together. This enhances the students’ sense of participation and makes them feel trusted. It also helps them realize their value in the team. (E1); My teaching demonstration is not only a demonstration of technology but also a process of exploring and solving problems with students. I try to let the students participate in the demonstration. After the demonstration, I always advise students to actively explore the purpose of doing so rather than simply imitating. (E3); When teaching dramatic vocal pieces, I use more dramatic and dynamic demonstrations to inspire students to perform. Being student-centred and encouraging them to participate. (E4)*
Focus on the process, not the outcome	*In order to avoid students thinking that the lecturer’s demonstrations are ‘out of reach,’ I will deliberately emphasize the importance of effort and practice. I will tell the students about my own experience, for example, I had a lot of difficulties at the beginning when I learnt this piece, but I believed that I could do it and persevered. Practice every day instead of hurrying. Students will learn that success is not something that that you are born with, but something you achieve through repeated effort. (E3); Some students observing my demonstration would say ‘Teacher, you are so good, I do not think I can do it.’ At this point, I would tell them ‘The key is not the result, but the step-by-step process of getting there.’ (E4); Students often think that teachers’ demonstrations are inherently perfect performances, but I will deliberately show them the effort. I always remember my students telling me ‘your demonstrations and sharing made me realise that successful performances are practised, not something perfect from the start, and that gave me more confidence to keep going.’ (E5).*
Error-based learning	*I will first demonstrate the movement in an incorrect way, such as using stiff hands or moving too quickly, and then demonstrate it again in the correct way. Then I will ask students: “Can you see the difference? What needs to be adjusted?” By observing and comparing, students can more easily understand key points of movement and reduce the fear of making mistakes. (E6); Many students are overly sensitive to their mistakes when singing and are afraid that the mistakes will affect the entire choir. In order to make them understand that mistakes are part of learning, I will intentionally conduct “failure demonstrations”. Let them know that even teachers make mistakes, so mistakes are not that scary and can be corrected through practice. (E8)*

The participants noted the importance of being student-centred and encouraging their active participation in observation and experimentation. Educators encourage students to repeat their demonstrations or lead other peers in practice, As Educator E1 mentioned, this enhances student engagement and makes them feel trusted. Educator E3 also pointed out that it is not only a demonstration of technology, but also a process that encourages students to explore and solve problems together. Participants also emphasised effort and progress rather than focusing on perfect results. They observed that during their demonstrations they would tell the students that successful demonstrations do not happen overnight but are achieved through long-term practice. Educator E3 shared his own difficulties and experiences in practicing and his perseverance encouraging the students to believe that they can succeed and should keep working hard.

The respondents concluded by encouraging the students to learn from the failures they observed. They also made deliberate mistakes and then corrected them. This was done in order to show ways that mistakes are commonly made, and then the educator showed the correct method, prompting students to analyse and adapt their own approach. This approach reduces the fear of failure and the notion of improvement based on the number of attempts taken, giving students more assurance that they can deal with challenges.

### Verbal persuasion and feedback practices

Verbal persuasion is one of the sources of self-efficacy according to Bandura’s theory. Positive verbal encouragement can be effective in increasing self-efficacy. Educators were asked *how verbal encouragement can be used to increase prospective music teachers’ self-efficacy.* The interviewees stated that singing classes give students enough opportunities to participate in practice, so it is important to evaluate the students’ performance. Respondents emphasised that it is crucial to approach feedback from an encouraging perspective, providing students with positive, constructive evaluations to promote and improve their self-efficacy.

Table 5 shows the strategies educators use to increase the self-efficacy of prospective music teachers through verbal persuasion. Respondents indicated that verbal persuasion is commonly used to increase self-efficacy in the classroom, rehearsals, and performances. These strategies can be divided into three categories: constructive feedback system, targeted and individualised affirmation, and fostering a supportive environment. Educators emphasised the importance of having the right feedback mechanisms in place, for example, Educator E7 stated that students should set achievable goals, Educators E1 and E3 highlighted the importance of timely praise when students make progress, and Educators E4 and E5 added that focusing on the process helps students understand that success is not achieved overnight, but rather through consistent and diligent effort over time.

**Table 5:** Strategies for promoting self-efficacy through verbal persuasion.

Themes	Statements of respondents
Constructive feedback system	*When students make breakthroughs in their practice, I immediately provide positive feedback, such as: ‘Your improvement in pitch today is very noticeable. I am touched by your hard work and proud of your progress.’ (E1); Students inevitably feel nervous in front of an audience, and as lecturers, we need to empathise with them. I often say to them, ‘I know this performance is challenging, but you need to remember that you have worked extremely hard, and that is truly admirable. You do not need to rush towards perfection—just show what you have practised, and it will be great!’ (E4); Progress in learning does not happen overnight. I will constantly remind my students of the importance of consistent effort. I will also give them encouragement when they notice progress, so that they are motivated to keep practising. I will emphasise their hard work and dedication to help them realise that success is not an accident. (E5); I will set achievable short- and long-term goals with my students and recognise them when they reach them. (E7)*
Targeted and individualised affirmation	*Each student’s learning pace and challenges are different, and I will provide individualised encouragement for each student’s unique performance. (E2); I focus a lot on giving specific feedback based on each student’s individual performance rather than using common words of encouragement. This makes the students feel cared for and knows exactly what they have done well, which increases their confidence. (E3); I will incorporate the experiences of different composers in order to inspire the students. I will tell them about the background of the composer’s work and the spirit of not giving up even in difficult times. Encourage them to learn from the composers and work consistently. (E4); I will also share my own experiences in the class, for example, I had a similar difficulty in the learning process, but through constant practice and the correct breathing method, I eventually overcame it. Afterwards, I encourage students to persevere and let go of fear of failure and say that I believe they can do it too. (E6)*
Fostering a supportive environment	*When a student makes progress, I will take the other students with me to applaud him. This not only makes him more confident, but also increases the motivation of the other students, as they see that they are encouraged and praised when they perform well. (E1); I will pay special attention to students’ progress in the learning process and recognise even small improvements. Recognition of progress allows students to realise that even though the learning process may be slow, every little effort is worth it. (E5); In addition to my own encouragement, I also help the students encourage each other. The goal is to create a positive learning atmosphere throughout the entire class, where students can feel their value and progress through the support of their peers. (E7); Creating a supportive classroom atmosphere is essential to build students’ confidence and teamwork. During rehearsals, I encourage positive interaction and mutual support among students, urging them not only to focus on themselves but also on each other to help everyone grow. (E8)*

The participants mentioned the importance of targeted and individualised affirmations for self-efficacy. Emphasis was placed on specific student achievements rather than general praise. As pointed out by Educator E2, each student has a different learning pace and needs targeted affirmations to help students recognise their progress, and Educators E3 and E8 also mentioned that pointing out specific details of what a student has done well and giving positive feedback instead of using common words of encouragement makes students truly cared for and recognised. This is not only verbal encouragement, but also emotional communication, which helps students build up a deeper sense of self-confidence and allows them to express themselves more courageously. Educators E4 and E6 used their personal experiences and background as composers and encouraged students to persevere and believe that through hard work. Through these different ways of encouragement, teachers helped the students build their self-efficacy in their studies and made them feel supported and cared for, so that they would not give up easily when faced with more challenges and would have more confidence and motivation to cope with them.

The other common practice of educators is to create a positive and supportive environment, particularly when it comes to group work. Educator E8 stated the necessity of a positive atmosphere during the rehearsal to unite all the people in a choir, encouraging students to help each other and interact positively. Such a positive and stress-free environment makes students feel safe and respected, which is essential for building self-confidence and increasing teamwork. Educator E5 stated that giving encouragement to students for even small improvements makes them realise that their efforts are worthwhile. The educator encourages students to face challenges and provides continuous support, emphasising the process of effort rather than the pursuit of a perfect result. Educators E1 and E7 mentioned that not only do they encourage the students themselves, but they also bring the rest of the class together to applaud those who do well. Students receive affirmation from the teacher and peers, increasing their self-efficacy. Finally, the teacher educator stressed that feedback should be a two-way dialogue, involving questions about what students are doing, thinking and feeling. It is important to ensure that feedback supports students’ self-efficacy without undermining their confidence.

### Regulating emotional/physiological states

Bandura’s theory of self-efficacy states that emotional and physiological states are also important factors affecting self-efficacy. In the context of music, prospective music teachers face numerous challenges that one goes through like the pressure to learn hard music pieces, daily practice and performing or presenting in front of the audience, this necessitating them to be in a good mood in order to handle the pressure. Educators were asked *how they help prospective music teachers maintain positive emotional and physiological states.*

The respondents highlighted that psychological issues, such as tension and anxiety, are common at different stages of development for prospective music teachers. They need the support of society, family, peers and lecturers. However, educators often struggle to recognise the psychological states of their students, emphasising the importance of providing more care and communication.

Table 6 categorises the strategies used by the educators to strengthen the positive emotional and physiological states of the students into five main subcategories: understanding individual needs, stress management, emotional regulation, emotional connection to music and physical preparation. Understanding of individual needs of students was mentioned most frequently, that is, genuine concern for the ideas and needs of students. Educator E1 and E8 highlighted that each student is unique and should receive personalised support and guidance tailored to their individual needs and characteristics. Empathising with students, actively listening to their thoughts and concerns, and giving them your full attention and understanding are essential practices. Educators E2 and E5 noted that the additional time spent in conversations facilitates this strategy. Listening to the students helps establish trust with them and make them feel respected and safe, which is critical to establishing self-efficacy.

**Table 6:** Strategies for promoting positive emotional and physiological states.

Themes	Statements of respondents
Understanding students’ individual needs	*Each student feels and behaves very differently in class. In order to help them maintain a positive emotional and physical state, I focus not only on technical improvement but also on students’ psychological state and classroom experience. (E1); I try to guide my students in a more authentic and flexible way from their point of view. I will spend more time in dialogue with my students to find out how they feel about the class, their learning, and life difficulties. (E2); I pay close attention to how my students are doing in the classroom. If I notice that certain students are becoming anxious because the content is too difficult, I will take the initiative to adjust the tempo and give more time to practice the easier content (E4); I will specifically talk to the students one-on-one, listen to them carefully, and understand the worries and difficulties they face. This will help them solve their problems and keep them positive. (E5); I focus on the individual differences of the students and understand their needs to tailor the instruction to the characteristics of different students. When I put myself in their shoes, the students will feel understood and respected and the teacher-student relationship will be more harmonious. They will also be more willing to participate in rehearsals and other activities. (E8)*
Stress management	*Many students indicated that I taught them some stress management techniques such as deep breathing, meditation, and positive thinking exercises that helped them stay focused and calm while practising and reduced their anxiety. (E1); Music itself is a powerful stress-relief tool. I would recommend that students listen to some relaxing music while studying or practising or enjoy their favourite music in their spare time. (E2); I often remind students to manage their time effectively to reduce the stress caused by procrastination or overloading. (E3); People will be relatively uneasy in unfamiliar environments. I will ask students to rehearse as many times as possible before performing on stage to effectively help reduce performance anxiety. (E4); I invite mental health experts to give talks to help students understand the importance of mental health and how to recognise and cope with stress. (E6); I will design improvisation exercises where they can complete an extension or* var*iation of a melody in their own way. There is no right or wrong way to do these exercises, the point is for students to find the confidence to express themselves musically and to reduce their anxiety. (E7); I organise group discussions in which students share their sources of stress and coping strategies. Through this interaction, they can not only feel the understanding and support of their peers but also gain new coping methods from others. Many students mentioned that this kind of communication made them feel less alone and more easily faced the challenges in their studies and life. (E8)*
Emotional regulation	*I always encourage students to try boldly and tell them that “failure is the mother of success. Do not be afraid when you make a mistake, but think and reflect calmly. Try not to make the same mistake next time, and you will succeed. (E2); It is important to express emotions appropriately. Negative emotions can be ventilated by confiding in others, writing in a diary or creating art. For example, when you feel depressed, you can talk to a friend or record your inner feelings by writing, which not only releases your emotions but also helps to clear your mind and find a solution to the problem (E5); Changing the environment can also be an effective way to regulate mood. Sometimes I will move my class to a park or to a sunny room. The natural environment can bring a sense of tranquillity and help relieve stress and anxiety. (E6)*
Emotional connection to music	*The emotional content of the songs to connect students with the material, helping them engage more deeply. Students have given me feedback that practising becomes more meaningful and enjoyable when they have an emotional connection to the music, which helps them stay emotionally positive. (E3); Many students become nervous when singing on stage or having technical difficulties. I will guide them through the emotional expression of the song to divert excessive focus to the technique. For example, when practising a lyrical art song, I will lead the students to deeply interpret the emotional connotation of the lyrics first and then drive the breakthrough of vocal technique through emotional expression. (E4); The use of multimedia resources such as video and audio can enrich the classroom experience and help students better understand musical works. For example, by watching videos of relevant performances or listening to the expressions of different performers, students are able to more intuitively feel the emotional expression of the music. (E6)*
Physical preparation	*The importance of a healthy diet, moderate exercise, and adequate sleep, all of which help students stay in a better physical state. (E1); Choosing suitable background music for warm-up activities can effectively stimulate students’ participation and motivation. For example, playing relaxing and rhythmic music during warm-up encourages students to perform simple body movements, such as stretching, twisting, and easy dance movements, in time with the beat of the music. Such activities can help students relax their bodies and enhance the atmosphere of the classroom, making it easier for them to enter the learning mode. (E3); It is especially important to have a vocal warm-up, especially in vocal lessons. The vocal cords and respiratory system can be activated through simple scale exercises, long tone exercises or oral exercises. For example, I can guide my students to ‘humming’ or ‘sounding out vowels,’ which not only helps to relax the laryngeal muscles, but also improves vocal flexibility and control. (E4); Rhythm is crucial for a choir. Using physical movements to enhance the students’ sense of rhythm is an effective warm-up technique. I lead the students through simple rhythmic exercises, such as clapping or stepping, to help them internalise the beat of the music. (E7)*

In the area of stress management, educators shared a variety of effective ways to help students reduce anxiety. Educator E1 mentioned using techniques such as deep breathing and meditation to help students relax, while Educator E2 pointed out that ‘music itself’ is a good stress relief tool and suggested that students relieve stress listening to music they like. Educator E4 invited mental health experts to provide guidance on stress management to students, which can help students better cope with stressors in their studies and lives. Educator E8 mentioned that by allowing students to share each other’s coping strategies through group discussions, it can not only establish a mutually supportive learning atmosphere, but it can also make students feel that they are not alone, thereby increasing their confidence in coping with challenges.

Emotion regulation focuses on teaching students how to manage and express emotions in positive ways. Prospective music teachers are under pressure to learn and perform, as well as teach in the future, and mood swings are inevitable. How to adjust in times of negative emotions is an important part of promoting their mental health and academic success. Educator E2 and E4 both stated that they encouraged students not to be afraid of making mistakes, not to be frustrated by mistakes but to be calm and learn from them, and not to overvalue the results but to emphasise the importance of the process of working hard. Educator E5 suggested that students find appropriate outlets to express their emotions, such as writing in a journal or confiding in friends and family. Educator E6 fully agreed and added that changing the environment is also an effective way to regulate emotions.

The emotional connection to music is the relevant method to make students learn the process of learning music and enjoy it at a more recent level, and in that way, it is an important strategy to improve their understanding and motivation. Educator E3 shared that by guiding students to explore the emotional connotations of the songs, the practice becomes more meaningful and enjoyable. Educator E4 observed that students sometimes focus too much on the technical aspects and neglect the emotional expression, so he led the students to interpret the emotional connotation of the lyrics first, and then push the breakthrough of vocal technique through emotional expression, while Educator E6 mentioned that the use of multimedia technology, such as video and audio, enriched the content of the classroom, and also helped students intuitively understand the expression of the emotions of the musical works. The participants indicated that this emotional connection improved the students’ musical expression and reduced their anxiety about technical challenges.

Finally, respondents indicated that a good physical condition is the foundation of everything. The importance of preparatory physical activities was emphasised. Educator E1 promotes a healthy lifestyle. In music teaching, warm-up movements not only help students activate their physical state but also improve their mood and concentration. The educators will lead the students through a simple physical and vocal warm-up before formal singing. For example, Educator E3 mentioned simple body movements accompanied by rhythmic music such as stretching, twisting, and easy dance movements, and Educator E7 led students to perform simple tempo exercises such as clapping or stepping before rehearsals according to the choir’s characteristics for the need for tempo. Body movements are used to help them internalise the music and enhance students’ sense of tempo. Educator E4, as a vocal educator, emphasises the importance of a vocal warm-up. These help prospective music teachers develop good habits and maintain a healthy and active state to cope with various challenges.

Inductive analysis of participants’ responses to the six interview prompts identified six themes central to the development of prospective music teachers’ self-efficacy: definition & understanding, influencing factors, task design, educator modelling, verbal encouragement, and emotional regulation. These themes provide practical guidance for fostering self-efficacy in teacher-education contexts.

## Discussion

This research aimed to provide insights into the processes of building self-efficacy among prospective music teachers in the Chinese higher education system in the light of the views of experienced university educators. The results contribute to a body of literature growing on self-efficacy as a primary psychological construct underlying successful teacher education and professional practice (Bandura, 1977; Schunk and Pajares, 2002; Gill et al., 2022). Although the role of self-efficacy in general teacher education is clear based on previous research (Tschannen-Moran and Hoy, 2001; Michaud, 2023), this study expands the concept to the situation of singing-related activities in the training of music teachers. This context often involves emotional expression, ensemble collaboration, and performance anxiety, which have been under-examined in relation to efficacy development (Osborne and McPherson, 2018; Hallam, 2010; Hendry et al., 2022).

The conclusion made by all the educators in the interviews was that self-efficacy is a central factor in prospective music teachers’ skills to learn, perform, and teach. Respondents noted that students with high self-efficacy were more willing to persevere in practice, find solutions when faced with technical difficulties, and demonstrated higher levels of emotional regulation and resilience in the teaching and learning process. Respondents agreed that classroom practice-teaching is the best way to increase self-efficacy among prospective music teachers, and Schunk and Pajares (2009) also indicated that practice-teaching experience is a determining factor in educators’ self-efficacy. Respondents indicated that prospective music teachers must have solid professional skills or they will have difficulty building confidence in their teaching, and Concina (2023) study also indicated that the acquisition of music skills is highly self-efficacy related, and that prospective teachers’ skills to perform, conduct and deliver choral music training has a direct impact on their confidence in the classroom. The respondents emphasised that feedback from tutors and peers can greatly enhance students’ confidence in teaching. Students’ belief that “I can do it too” can be strengthened by observing the success stories of their peers (Bandura, 1977). The respondents noted that music teachers must learn to manage stress and adapt to different teaching environments. The study by Biasutti and Concina (2017) suggests that self-efficacy in music education is closely related to stress management skills and appropriate regulation of emotions reduces anxiety in the classroom and improves teaching quality.

Based on the results of the interviews, the educators proposed a variety of strategies to promote self-efficacy among prospective music teachers: (1) the educator adopted the ‘simple to complex’ teaching strategy to help students gradually build up their confidence; (2) the educator demonstrated the correct playing techniques and asked students to imitate them so as to nurture their self-confidence; (3) the educator adopted the ‘peer teaching’ model to allow students to coach each other and enhance their sense of teamwork; (4) the educator encouraged students to record their classroom performance and review and analyse it after the class. Although identified within Chinese higher music education, these strategies map onto Bandura’s four sources of self-efficacy (mastery, vicarious experience, verbal persuasion, physiological/emotional states) and are therefore mechanistically generalisable beyond China. Their enactment, however, depends on assessment stakes and classroom norms (e.g., how public correction and peer feedback are used), so context-sensitive adaptation is warranted.

The study is based on the music education system of the China Normal University, but the results may not apply to other countries or regions. Findings reflect educators’ perspectives (single-source, indirect data); future work will triangulate with student self-reports/interviews and naturalistic classroom observations to corroborate and extend these process accounts. To capture development more directly, future studies should adopt longitudinal, observational, mixed-methods designs: combine repeated student self-efficacy measures (e.g., pre-mid-post semester and pre/post practicum) with video-based observation and coding of micro-teaching and juries mapped to Bandura’s four sources, plus brief diary/experience-sampling of arousal and feedback. Linking these time-varying exposures to within-person change would triangulate and extend the educator-reported processes. The study focused on the specific university and classrooms and could be extended to a wider range of educational settings in the future. Although the music education system of the China Normal University was the basis for the study, the development of self-efficacy of prospective music teachers can vary between cultures. Future research could make cross-national comparisons to explore the effects of different educational systems on prospective music teachers’ self-efficacy. Digital technologies such as online teaching platforms, artificial intelligence feedback systems, and virtual-reality tools of music-teaching related to self-efficacy of music teachers are also the resources that can be used in the context of the study of how the process of approaching the music teacher self-efficacy is informed and impacted by such advances. This study refines how Bandura’s four sources operate in the Singing lecture, extending the framework’s applicability to music at the mechanism level without altering its core.

## Conclusion

Research advances the understanding of how self-efficacy is cultivated in prospective music teachers within the unique context of Chinese higher music education. Drawing on the reflective expertise of experienced university educators, the study demonstrates that the effective development of self-efficacy depends not only on the mastery of musical skills, but also on deliberate pedagogical strategies that support emotional regulation, collaboration, and adaptability. These findings highlight the need to integrate self-efficacy enhancement into music teacher training programmes to prepare future educators for the complex challenges of modern classroom and performance environments. The research contributes to theory and practice by offering concrete recommendations to promote self-efficacy through singing-related coursework. Moving forward, continued research in diverse educational contexts will be essential for developing comprehensive models of teacher education that address the evolving demands of music education worldwide.

## Data Availability

The raw data supporting the conclusions of this article will be made available by the authors, without undue reservation.

## Appendix S1

Semi-structured interview guide (6 items).

This appendix lists the six core prompts used in the semi-structured interviews. Questions were asked flexibly with follow-up probes as needed.

1. How educators define and understand the concept of self-efficacy in prospective music teachers?
2. Based on the educators’ experiences, the factors that most influence the self-efficacy of prospective music teachers?
3. How they design tasks to help prospective music teachers build self-efficacy during their lectures (Solfeggio, Vocal, Chinese Opera, Choir)?
4. How they promote prospective music teachers’ self-efficacy by allowing them to observe the educators’ modelling in their courses?
5. How verbal encouragement can be used to increase prospective music teachers’ self-efficacy?
6. How they help prospective music teachers maintain positive emotional and physiological states?

Note. These prompts align one-to-one with the six subsections in the Results.
